# Protein proximity networks and functional evaluation of the casein kinase 1 gamma family reveal unique roles for CK1γ3 in WNT signaling

**DOI:** 10.1016/j.jbc.2022.101986

**Published:** 2022-04-27

**Authors:** Megan J. Agajanian, Frances M. Potjewyd, Brittany M. Bowman, Smaranda Solomon, Kyle M. LaPak, Dhaval P. Bhatt, Jeffery L. Smith, Dennis Goldfarb, Alison D. Axtman, Michael B. Major

**Affiliations:** 1 Lineberger Comprehensive Cancer Center, University of North Carolina at Chapel Hill, Chapel Hill, North Carolina, USA; 2 Department of Pharmacology, University of North Carolina at Chapel Hill, Chapel Hill, North Carolina, USA; 3 Department of Cell Biology and Physiology, Washington University in St Louis, St Louis, Missouri, USA; 4 Division of Chemical Biology and Medicinal Chemistry, Structural Genomics Consortium, UNC Eshelman School of Pharmacy, University of North Carolina at Chapel Hill, Chapel Hill, North Carolina, USA; 5 Institute for Informatics, School of Medicine, Washington University in St Louis, St Louis, Missouri, USA; 6 Department of Otolaryngology, School of Medicine, Washington University in St Louis, St Louis, Missouri, USA

**Keywords:** WNT signaling, kinase inhibitor, protein kinase, casein kinase 1 gamma, proteomics, ACN, acetonitrile, AKI, AKI00000062a, APC, adenomatous polyposis coli, ATCC, American Type Culture Collection, CELSR2, cadherin EGF LAG seven-pass G-type receptor 2, CK1α, casein kinase 1α, CM, conditioned media, CSNK, casein kinase, DVL, disheveled, FA, formic acid, FDR, false discovery rate, FP1, FP1-24-2, FZD, Frizzled, GSK3β, glycogen synthase kinase 3β, HEK293T, human embryonic kidney 293T cell line, IDR, intrinsically disordered region, LRP6, low-density lipoprotein receptor–related protein 6, MS, mass spectrometry, mT, miniTurbo, NIH, National Institutes of Health, PIK3R3, phosphoinositide-3-kinase regulatory subunit 3, PoC, Percent of Control, RIPA, radioimmunoprecipitation assay, RIPK, receptor-interacting serine∖threonine kinase, SAINT, Significance Analysis of INTeractome, SOD1, superoxide dismutase 1, W. blot, Western blot, ZDHHC8, zinc finger DHHC-type palmitoyltransferase 8.

## Abstract

Aberrant activation or suppression of WNT/β-catenin signaling contributes to cancer initiation and progression, neurodegeneration, and bone disease. However, despite great need and more than 40 years of research, targeted therapies for the WNT pathway have yet to be fully realized. Kinases are considered exceptionally druggable and occupy key nodes within the WNT signaling network, but several pathway-relevant kinases remain understudied and “dark.” Here, we studied the function of the casein kinase 1γ (CSNK1γ) subfamily of human kinases and their roles in WNT signaling. miniTurbo-based proximity biotinylation and mass spectrometry analysis of CSNK1γ1, CSNK1γ2, and CSNK1γ3 revealed numerous components of the β-catenin–dependent and β-catenin–independent WNT pathways. In gain-of-function experiments, we found that CSNK1γ3 but not CSNK1γ1 or CSNK1γ2 activated β-catenin–dependent WNT signaling, with minimal effect on other signaling pathways. We also show that within the family, CSNK1γ3 expression uniquely induced low-density lipoprotein receptor–related protein 6 phosphorylation, which mediates downstream WNT signaling transduction. Conversely, siRNA-mediated silencing of CSNK1γ3 alone had no impact on WNT signaling, though cosilencing of all three family members decreased WNT pathway activity. Finally, we characterized two moderately selective and potent small-molecule inhibitors of the CSNK1γ family. We show that these inhibitors and a CSNK1γ3 kinase–dead mutant suppressed but did not eliminate WNT-driven low-density lipoprotein receptor–related protein 6 phosphorylation and β-catenin stabilization. Our data suggest that while CSNK1γ3 expression uniquely drives pathway activity, potential functional redundancy within the family necessitates loss of all three family members to suppress the WNT signaling pathway.

WNT signaling regulates embryonic development, injury repair, regeneration, and tissue homeostasis (1, 2). WNT signaling mechanisms are tightly controlled by feedback loops and rapid protein degradation. In disease states including cancer, neurodegeneration, and bone density disorders, regulatory mechanisms governing WNT signaling are aberrantly active or inhibited (3, 4). In some cancers, mutations in core signaling proteins are common, including adenomatous polyposis coli (APC) mutations in greater than 80% of colorectal adenocarcinoma. In other cancers of the lung and breast, increased WNT ligand secretion from stromal cells drives pathway activity (3, 4). While WNT signaling has been well studied across many disease states and several therapeutics have been developed, targeting WNT clinically remains an unmet need (1, 3, 5). Identifying and understanding new regulators of WNT signaling may reveal therapeutic targets to improve patient outcome for WNT-associated diseases.

Kinases, a highly druggable class of proteins, are master regulators of various signaling pathways, including WNT signaling. WNT/β-catenin-dependent signaling is often described with respect to the main effector protein, β-catenin. In the absence of WNT ligand, a destruction complex comprised of casein kinase 1α (CK1α; casein kinase 1A1 [CSNK1A1]), glycogen synthase kinase 3β (GSK3β), AXIN1/2, and APC phosphorylates β-catenin resulting in its subsequent ubiquitylation and degradation by β transducin repeat–containing protein (β-TRCP) and the proteasome (6, 7, 8). In the presence of WNT ligand, the WNT receptors low-density lipoprotein receptor–related protein 6 (LRP6) and Frizzled (FZD) co-complex, and an alternate signaling complex, the WNT signalosome, forms around the intracellular tail of LRP6. Signalosome formation transiently suppresses β-catenin phosphorylation, allowing β-catenin to accumulate, translocate to the nucleus, and drive transcription of WNT response genes (9, 10, 11). Several kinases (CSNK1 family, GSK3β, receptor-interacting serine∖threonine kinase 4 [RIPK4], adaptor-associated protein kinase 1 [AAK1], the GRK family) regulate WNT signaling in both the signalosome and the destruction complex (12, 13, 14, 15, 16, 17). Of these kinases, GSK3β has been the most thoroughly studied within the WNT field and as a master regulator of cell signaling networks (12, 18). The CSNK1 family (referred to as CK1 hereafter), while also widely studied, is more complex. The CK1 family is comprised of CK1α (CSNK1A), CK1δ (CSNK1D), CK1ε (CSNK1E), and CK1γ (CSNK1G). CK1α functions almost exclusively within the destruction complex where it phosphorylates β-catenin to prime for phosphorylation by GSK3β (8). This triggers the ubiquitylation and degradation of β-catenin. CK1δ and CK1ε are thought to function redundantly within the destruction complex and signalosome (19, 20). The CK1γ subfamily, as well as CK1δ and CK1ε, have a reported role in priming LRP6 for GSK3β phosphorylation and subsequent signalosome formation (19, 20).

The CK1γ subfamily of kinases is comprised of three genes: CK1γ1 (CSNK1G1), CK1γ2 (CSNK1G2), and CK1γ3 (CSNK1G3). All three are identified as understudied and dark kinases by the National Institutes of Health initiative, Illuminating the Druggable Genome (https://commonfund.nih.gov/idg/index) (21). The CK1γ subfamily share 92% sequence homology within their kinase domain. The homology in their C-terminal regulatory domain is 41% conserved, with CK1γ3 containing a unique 33 amino acid insertion (Fig. 1*A*). Studies suggest that the palmitoylation modification positions CK1γ kinases to act within the WNT signalosome, as other CK1 family members are not anchored to the membrane (19). Apart from an emerging role of CK1γ in WNT/β-catenin signaling, the CK1γ family has described roles in promoting phosphorylation of p65 (NF-κB subunit), resulting in inhibition of the retinoic acid–inducible gene 1–mediated signaling (22). In addition, CK1γ activates tumor necrosis factor alpha signaling (23). Within the CK1γ family, CK1γ2 has been shown to inhibit activation of RIPK3, resulting in suppressed RIPK3-driven necroptosis (24). Interestingly, superoxide dismutase 1 (SOD1) positively regulates CK1g3 expression and subsequent WNT activation (25). In *Drosophila*, Gilgamesh (CK1γ *Drosophila* homolog) functions within β-catenin-independent signaling to regulate Van Gogh phosphorylation at the cell membrane, as well as regulating Notch pathway activity (26, 27, 28).

**Figure 1:**
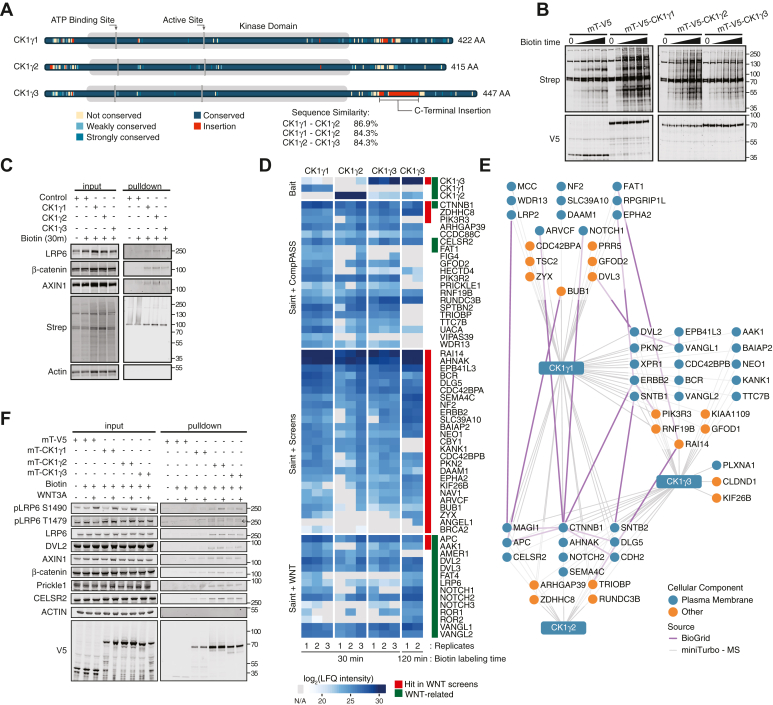
**CK1γ proximity-based interaction networks identify WNT components.***A*, schematic of CK1γ family highlighting conserved amino acids and sequence similarity between family members. *B*, W. blot analysis of HEK293T cells stably expressing mini-Turbo CK1γ or V5 control incubated with 50 mM of biotin over a time course (untreated, 15 min, 30 min, 45 min, 1 h, and 2 h). *C*, W. blot analysis of streptavidin affinity pulldown from stable HEK293T cells expressing mini-Turbo CK1γ or V5, treated as indicated. *D*, heat map demonstrating changes in log_2_(LFQ intensity) of protein proximity partners for the CK1γ subfamily. *Red track* indicates hits in previously published WNT screens (Tables S1 and S2). *Green track* indicates well-established proteins involved in WNT/β-catenin-dependent and WNT/β-catenin-independent signaling. *E*, streptavidin affinity purification mass spectrometry (APMS) proximity networks for CK1γ1, CK1γ2, and CK1γ. HEK293T cells stably expressing mT-V5-CK1γ were treated with 50 mM biotin for 30 min before streptavidin APMS. *Blue circle* indicates localization to the plasma membrane. *Gray lines* represent bait–prey interactions. *Purple lines* indicate prey–prey interactions collected from the BioGRID database. *F*, W. blot validation of mT-CK1γ protein–protein interaction networks treated with biotin and WNT3A CM for 30 min. CK1γ, casein kinase 1γ; CM, conditioned media; HEK293T, human embryonic kidney 293T cell line; LFQ, label-free quantitation; W. blot, Western blot.

Following WNT ligand–induced FZD–LRP6 co-complex, disheveled (DVL) is recruited to the intracellular tail of FZD (29). This binding promotes recruitment of the AXIN complex (GSK3β, CK1, and APC) to LRP6, forming the WNT signalosome (10, 30, 31). Central to WNT signalosome formation is phosphorylation of LRP6 at multiple sites, with GSK3β phosphorylating LRP6 at S1490 and CK1 family members phosphorylating LRP6 at T1479 and T1493 (19, 20). Ultimately, CK1 phosphorylation of LRP6 triggers AXIN recruitment (19, 20, 32, 33). Upon signalosome formation, GSK3β is sterically occluded by the cytoplasmic tail of phosphorylated LRP6, transiently suppressing β-catenin phosphorylation (10, 11, 19, 20). Though several studies have reported members of the CK1 family as essential regulators of LRP6 phosphorylation and establishment of the signalosome, several important questions remain. For example, each member of the CK1γ subfamily has not been individually evaluated within β-catenin–dependent WNT signaling. In addition, previously reported data relied exclusively on overexpression studies and presented no genetic or chemical loss-of-function data (19, 20).

Here, we defined protein–protein proximity networks for the CK1γ family and functionally evaluated their contribution to a small panel of disparate signaling pathways. Each CK1γ family member co-complexed with various WNT components, including β-catenin and core members of the planar cell polarity complex. Surprisingly, only CK1γ3 activated β-catenin-dependent WNT signaling when overexpressed. Our experiments reveal a family-specific role for CK1γ3 in promoting phosphorylation of LRP6 within the WNT signalosome. In addition, we characterized two pan-CK1γ–specific inhibitors. Genetic or chemical inhibition of the CK1γ family suppresses WNT signaling, but suppression of CK1γ3 alone had no effect. This finding was extended and confirmed with a kinase-dead CK1γ3 mutant, which suppressed WNT signaling.

## Results

### Physical and functional evaluation of the CK1γ family

To better understand each member of the CK1γ family, we defined their protein–protein proximity networks and functionally evaluated their impact on signal transduction. We used the miniTurbo (mT) promiscuous biotin ligase to comprehensively map CK1γ proximal proteins, which requires a shorter biotin labeling window compared with BirA∗ (34, 35). Specifically, in the presence of exogenous biotin, a protein of interest fused to the mT biotin ligase will result in biotinylation of surface-exposed lysine residues on proximal proteins, enabling their affinity purification with streptavidin and identification by Western blot (W. blot) or mass spectrometry (MS). Human embryonic kidney 293T (HEK293T) cells stably expressing mT-CK1γ1, mT-CK1γ2, and mT-CK1γ3 were treated with biotin for varying amounts of time before W. blot analysis for biotinylated proteins and for a V5 epitope, the latter of which is expressed in frame with mT (Fig. 1*B*). Streptavidin affinity purification followed by W. blot analysis confirmed proximal complex formation between CK1γ family members and WNT pathway proteins LRP6, AXIN1, and β-catenin (CTNNB1) (Fig. 1*C*). Next, in biological triplicate, we treated the stable cell lines with biotin for 30 min before streptavidin purification and MS analysis. Resulting identifications were probabilistically scored against controls with Significance Analysis of INTeractome (SAINT) and then further ranked with CompPASS before visualization (Table S1, sheet *B* and C) (36, 37, 38). Heat map representation of protein abundance revealed concordant proximal networks across the CK1γ family (Fig. 1*D*). Of 548 high-confidence protein interactions for CK1γ1, CK1γ2, and CK1γ3, 326 proteins were seen with all subfamily members. β-catenin, ZDHHC8 (zinc finger DHHC-type palmitoyltransferase 8), and PIK3R3 (phosphoinositide-3-kinase regulatory subunit 3) were the most abundant proximal proteins across all three networks. Several core components of the β-catenin-dependent and β-catenin-independent signaling pathway were also identified, such as DVL, APC, cadherin EGF LAG seven-pass G-type receptor 2 (CELSR2), and VANGL planar cell polarity protein 1 (Fig. 1*D*, *green track*). To further strengthen and expand the proximity networks, we repeated the experiment at 120 min of biotin treatment, revealing LRP6 and FZD3 as CK1γ3-proximal proteins (Fig. 1*D*). To further explore these networks and their connectivity to the WNT pathway, we annotated all proteins for functional contribution to β-catenin-dependent transcription. From four recently published independent genetic screens, 1143 genes were identified as functionally impactful to β-catenin-dependent transcription (Table S2) (39, 40, 41, 42). Integration of these data with the CK1γ proximity networks revealed 32 CK1γ proximal proteins that when silenced or CRISPR-deleted impacted β-catenin-dependent transcription (Fig. 1*D*, *red track*). Last, we visualized the data as a network to examine high-confidence prey–prey interactions and subcellular localization (Fig. 1*E*). The majority of the prey proteins are known to localize to the plasma membrane.

To confirm the MS results, and to test the impact of WNT3A stimulation, we analyzed the CK1γ affinity-purified proximal proteins by W. blot analysis (Fig. 1*F*). CK1γ2 and CK1γ3 interacted with β-catenin-dependent WNT signaling components (LRP6, DVL2, AXIN1, and β-catenin), whereas CK1γ1 interacted with these proteins but to a lesser extent. WNT3A stimulation increased pLRP6 S1490 in all three CK1γ pulldowns. Additional WNT3A-dependent changes were not observed. We also confirmed the interaction between the CK1γ family and proteins involved in β-catenin-independent signaling, CELSR2 and prickle planar cell polarity protein 1 (Fig. 1*F*).

**Figure 2:**
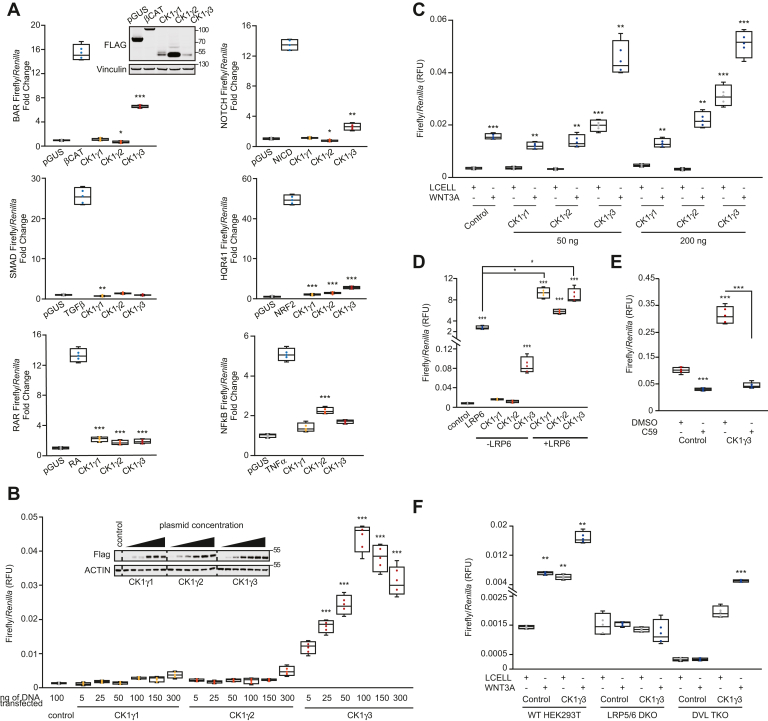
**Low-throughput reporter screen identifies CK1γ3 as an activator of WNT signaling.***A*, HEK293T cells were transiently transfected for 30 h with 100 ng of indicated positive control (β-catenin, NRF2, and NICD) construct or pGUS negative control or the indicated CK1γ construct. About 40 ng TK-*Renilla* and 40 ng of the indicated Response Element–Luciferase construct was included. Where indicated, cells were treated with (TGFβ 10 ng/ml, TNFα 10 ng/ml, and retinoic acid [RA] 10 ng/ml) for 6 h (TGFβ and TNFα) or 16 h (RA). Inset W. blot demonstrates protein overexpression for the BAR luciferase assay. Each condition was normalized to pGUS control. Error bars represent standard deviation, and statistics are compared to pGUS control. *B*, BAR luciferase assay from HEK293T B/R (stable BAR-firefly luciferase, TK-*Renilla*–expressing cell line) cells transfected for 24 h with indicated DNA mass. Statistics are compared to pGUS control. *Inset* blot is confirmation of overexpression for luciferase assay in *B*. *Dashed line* represents cropped blot for removal of redundant untreated sample. *C*, HEK293T B/R cells were transfected with the indicated construct for 14 h and then treated with Lcell or WNT3A CM for 16 h. All statistics are compared to FLAG-Control Lcell sample. *D*, HEK293T B/R cells were transfected with the indicated construct for 24 h before luciferase quantitation. Statistics are compared to pGUS control. *E*, HEK293T B/R cells were transfected with the indicated construct for 14 h and then treated with 10 μM C59 for 18 h. *F*, WT, LRP5/6 DKO, DVL TKO HEK293T cells transfected with 20 ng BAR-luciferase, 10 ng TK-*Renilla*, and 70 ng of indicated constructs for 14 h, and then treated with either Lcell CM or WNT3A CM for 18 h. Statistical significance presented is compared with Lcell-treated FLAG-Control cells for each cell type. For all panels: ∗∗∗*p* < 0.0005, ∗∗*p* < 0.005, and ∗*p* < 0.05. All data are plotted as box-and-whisker plots, with the boxes representing the median and the interquartile range. All biological replicates are averaged across three technical replicates. Statistical significance was evaluated by Student’s *t* test. CK1γ3, casein kinase 1γ3; CM, conditioned media; DKO, double KO; DVL, disheveled; HEK293T, human embryonic kidney 293T cell line; LRP, low-density lipoprotein receptor–related protein; NICD, Notch intracellular domain; NRF2, nuclear factor erythroid 2–related factor 2; TGFβ, transforming growth factor beta; TKO, triple KO; TNFα, tumor necrosis factor alpha; W. blot, Western blot.

We next functionally evaluated each member of the CK1γ subfamily across a small panel of engineered pathway-specific transcriptional reports. Transient transfection and overexpression of each CK1γ subfamily member in HEK293T cells revealed relationships to the WNT (BAR), Notch, transforming growth factor beta (SMAD), NRF2 (nuclear factor erythroid 2–related factor 2; hQR41), retinoic acid (retinoic acid receptor), and tumor necrosis factor alpha (NF-κB) signaling pathways (Fig. 2*A*) (15, 43, 44, 45). Surprisingly, only CK1γ3 activated the WNT reporter, with minimal effects from CK1γ1 and CK1γ2. Though statistically significant compared with the negative control, none of the CK1γ family members strongly regulated the other signal transduction reporters. To further establish the CK1γ3 selectivity for β-catenin-dependent activation of the BAR reporter, we performed a dose–response overexpression experiment. In contrast to CK1γ3, overexpression of CK1γ1 and CK1γ2 did not activate BAR (Fig. 2*B*). In the presence of WNT3A conditioned media (CM), CK1γ1 and CK1γ2 activated WNT signaling similar to control, whereas CK1γ3 overexpression increased activation of BAR (Fig. 2*C*) (19, 20). In addition, co-overexpression of LRP6 with either CK1γ1, CK1γ2, or CK1γ3 resulted in a significant activation of BAR compared with control and compared with LRP6 overexpression alone (Fig. 2*D*) (19, 20). Together, these data establish CK1γ3 as proximal to numerous WNT pathway proteins and as an activator of β-catenin-dependent WNT signaling. CK1γ1 and CK1γ2 share WNT pathway proximal proteins, but in the absence of co-overexpression of LRP6, they do not regulate β-catenin-dependent transcription.

### CK1γ3 requires WNT components to activate WNT signaling

To evaluate which WNT pathway proteins are required for CK1γ3 activation of β-catenin-dependent transcription, we studied established inhibitors of WNT signaling and KO cell lines. While overexpression of CK1γ3 activated BAR in the absence of WNT3A CM, treatment with C59, a PORCN inhibitor (required for WNT ligand palmitoylation and secretion), blocked this activation, which supports a role for autocrine WNT3A signaling in HEK293T cells (Fig. 2*E*) (46). We next tested the requirement of LRP6 and DVL. In contrast to HEK293T WT cells, overexpression of CK1γ3 in LRP5/6 double KO HEK293T cells did not activate WNT signaling (Fig. 2*F*). HEK293T cells lacking DVL1, DVL2, and DVL3 did not respond to WNT3A CM, as expected (47). Surprisingly, CK1γ3 overexpression increased BAR activity in DVL KO cells but to a lesser extent that in DVL WT cells (Fig. 2*F*). These data indicate that WNT3A ligand secretion and expression of LRP5/6 are required for CK1γ3-driven activation of β-catenin-dependent transcription; the DVL proteins are involved but are not absolutely required.

### CK1γ3 activates WNT signaling by increasing LRP6 phosphorylation

**Figure 3:**
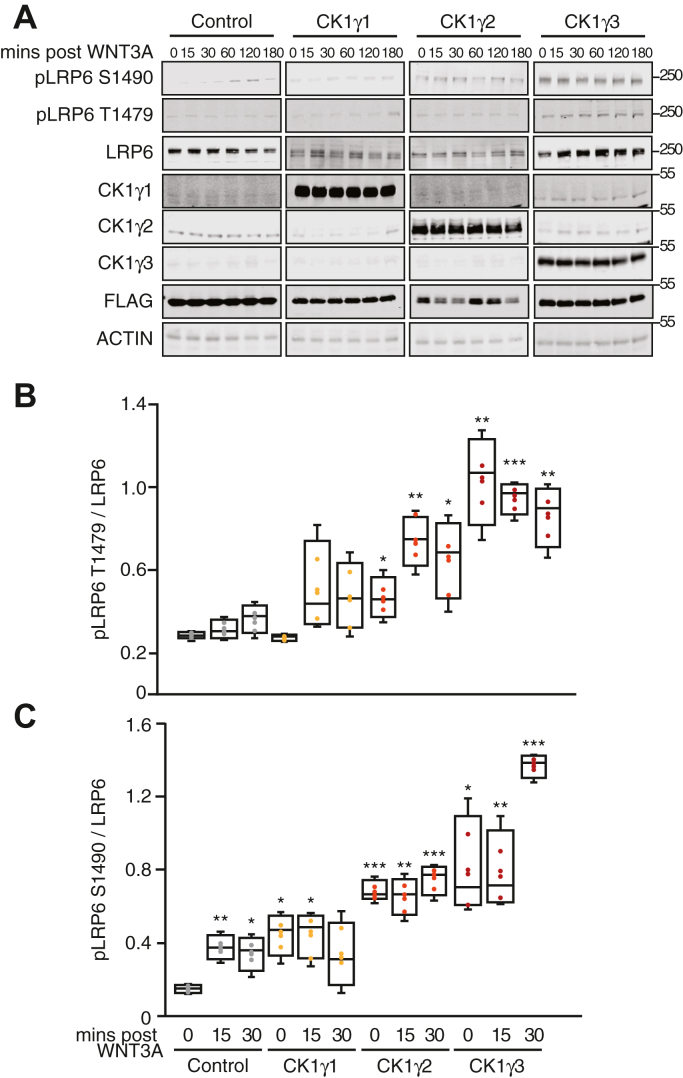
**Overexpression of CK1γ3 activates WNT signaling by increasing LRP6 phosphorylation.***A*, HEK293T cells were transfected with the indicated construct for 14 h and then exposed to WNT3A CM for indicated time and analyzed by W. blot. *B* and *C*, quantification of W. blots from *A* at indicated time points. pLRP6 T1479 (*B*) and pLRP6 S1490 (*C*) across four replicates, normalized to total LRP6. For all panels: ∗∗∗*p* < 0.0005, ∗∗*p* < 0.005, and ∗*p* < 0.05. All data are plotted as box-and-whisker plots, with the boxes representing the median and the interquartile range. All biological replicates are averaged across three technical replicates. Statistical significance was evaluated by Student’s *t* test. All statistics are compared to FLAG-Control untreated sample. CK1γ3, casein kinase 1γ3; CM, conditioned media; HEK293T, human embryonic kidney 293T cell line; LRP6, low-density lipoprotein receptor–related protein 6; W. blot, Western blot.

It was previously reported that overexpression of CK1γ1 together with overexpression of LRP6 and DVL resulted in LRP6 phosphorylation at T1493 and T1479 (19, 20, 48). In HEK293T cells, we tested whether overexpression of each member of CK1γ family impacted phosphorylation of endogenously expressed LRP6 (Fig. 3*A*). CK1γ1 overexpression did not increase LRP6 phosphorylation at T1479 or the GSK3β site, S1490, irrespective of WNT3A treatment (Fig. 3, *B* and *C*). With CK1γ2 overexpression, S1490 LRP6 phosphorylation increased in response to WNT3A but not T1479. CK1γ3 overexpression robustly increased LRP6 phosphorylation at T1479, as well as S1490, in both the absence and presence of WNT3A. Together, these data suggest that in contrast to CK1γ3, CK1γ1 and CK1γ2 do not activate WNT signaling in the absence of exogenous WNT3A ligand and do not induce LRP6 phosphorylation at T1479.

### CK1γ3 kinase activity is required for maximum activation of WNT signaling

**Figure 4:**
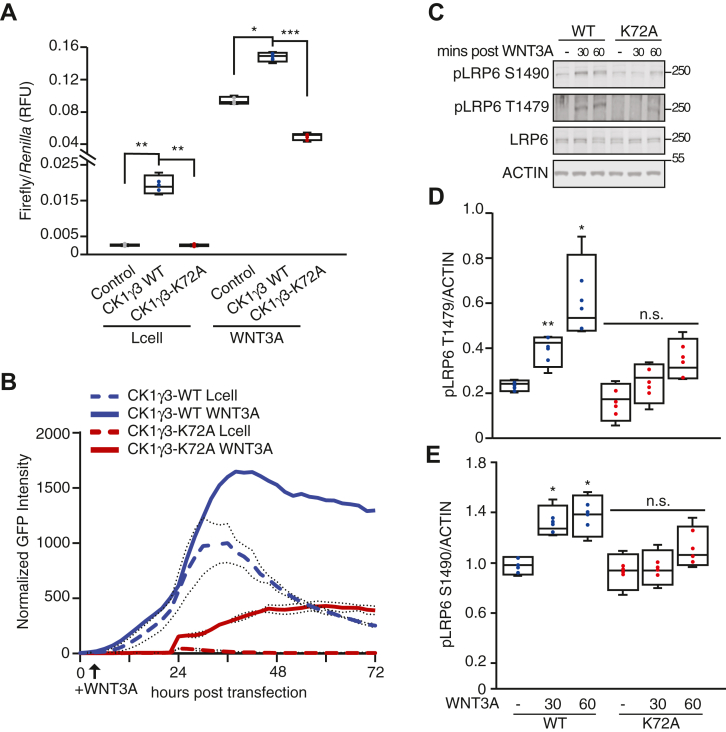
**CK1γ3 kinase activity is required for maximum WNT3A activation.***A*, HEK293T B/R cells were transfected with the indicated construct for 14 h, treated with Lcell or WNT3A CM for 18 h, and then analyzed by luciferase assay. *B*, live-cell imaging of HEK293T cells stably expressing a BAR-GFP fluorescent reporter transiently transfected with the indicated expression construct, CK1γ3-WT or CK1γ3-K72A. Lcell or WNT3A CM was added at 8 h, and cells were monitored for an additional 72 h. Data are averaged across four replicates. *C*, HEK293T cells transfected with either CK1γ3-WT or CK1γ3-K72A for 14 h then either untreated or treated with WNT3A CM for 30 or 60 min. Samples were then analyzed by W. blot. *D* and *E*, quantification of W. blot from *A* for indicated time points, pLRP6 T1479 (*D*) and pLRP6 S1490 (*E*) across four biological replicates, normalized to total LRP6. All statistics are compared to FLAG-CK1γ3 Lcell sample. For all panels: ∗∗∗*p* < 0.0005, ∗∗*p* < 0.005, and ∗*p* < 0.05. All biological replicates are represented in box-and-whisker plots, with the *boxes* representing the median and the interquartile range. All biological replicates are averaged across three technical replicates. Statistical significance was evaluated by Student’s *t* test. CK1γ3, casein kinase 1γ3; CM, conditioned media; HEK293T, human embryonic kidney 293T cell line; LRP6, low-density lipoprotein receptor–related protein 6; W, blot, Western blot.

We next performed loss-of-function studies to determine if the CK1γ family was required for WNT3A-driven β-catenin-dependent transcription. First, we generated a CK1γ3 kinase-dead mutant (CK1γ3-K72A). In contrast to CK1γ3-WT, expression of kinase-dead CK1γ3-K72A in HEK293T cells did not induce the activity of the BAR transcriptional reporter (Fig. 4*A*). In the presence of WNT3A CM, kinase-dead CK1γ3-K72A expression suppressed BAR activity as compared with WT CK1γ3 or control, which is supportive of dominant-negative activity (Fig. 4*A*). In an orthogonal experiment, CK1γ3 or CK1γ3-K72A was expressed in HEK293T cells stably harboring a BAR-GFP fluorescent reporter. Live cell imaging over 3 days revealed that CK1γ3-K72A suppressed WNT3A-driven β-catenin-dependent transcription (Fig. 4*B*). Last, we tested whether expression of the CK1γ3 kinase-dead mutant impacted the phosphorylation of LRP6. CK1γ3 overexpression resulted in LRP6 phosphorylation at both S1490 and T1479 sites following WNT3A CM exposure (Fig. 4, *C*–*E*). In contrast, overexpression of CK1γ3-K72A suppressed phosphorylation of LRP6 at both sites. These data demonstrate that kinase activity of CK1γ3 is required for phosphorylation of LRP6 and activation of the WNT pathway.

**Figure 5:**
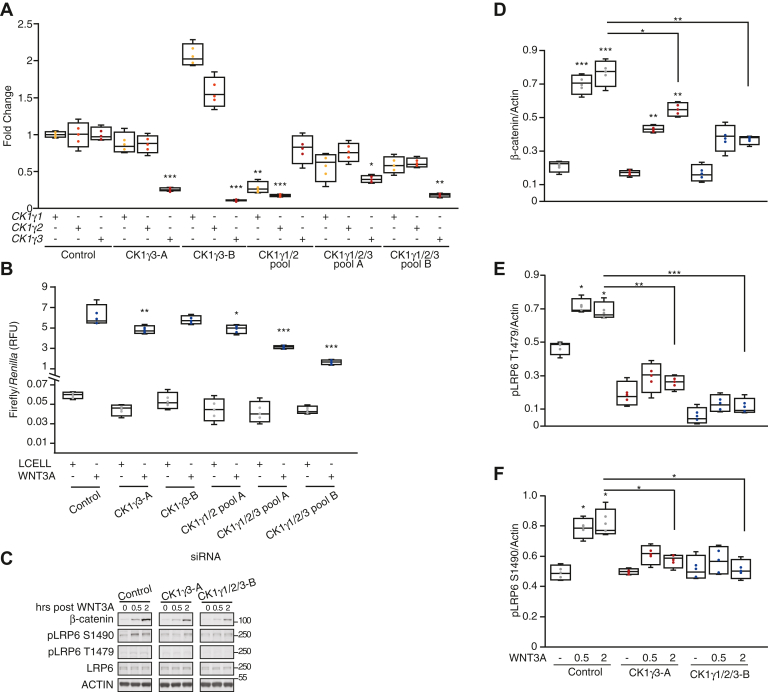
**Loss of CK1γ1/2/3 impairs WNT activation.***A*–*C*, RKO B/R cells were transfected with indicated siRNA or pooled siRNA for 48 h and then split for the following experiments: 24 h postsplit gene expression was analyzed by RT–qPCR (*A*), 6 h postsplit cells were treated with WNT3A for 18 h and then analyzed by luciferase assay (*B*), 24 h postsplit cells were exposed to a WNT3A time course, harvested at the indicated time post WNT3A, and analyzed by W. blot. For *A*, all statistics are compared to siControl expression for the specific gene. For *B*, all statistics are compared to siControl WNT3A-treated cells. *D*–*F*, quantification of W. blot from *E*, β-catenin (*D*), pLRP6 T1479 (*E*), and pLRP6 S1490 (*F*) across four replicates, normalized to actin. Statistics are compared to siControl untreated cells, unless otherwise stated. For all panels: ∗∗∗*p* < 0.0005, ∗∗*p* < 0.005, and ∗*p* < 0.05. All biological replicates are represented in box-and-whisker plots, with the *boxes* representing the median and the interquartile range. All biological replicates are averaged across three technical replicates. Statistical significance was evaluated by Student’s *t* test. CK1γ, casein kinase 1γ; qPCR, quantitative PCR; W. blot, Western blot.

To extend this finding, we tested the effect of CK1γ knockdown on BAR reporter activity. First, we confirmed knockdown by quantitative PCR of two nonoverlapping siRNAs for CK1γ3 as well as pooled siRNAs targeting CK1γ1/2/3 or CK1γ1/2 (Fig. 5*A*). Transfection of the indicated siRNA into HEK293T cells stably harboring BAR revealed that knockdown of CK1γ3 alone or a pool of CK1γ1/2 did not significantly inhibit WNT activation. However, knockdown of CK1γ1/2/3 suppressed WNT activation by approximately 50% (Fig. 5*B*). Second, we examined β-catenin protein stabilization and LRP6 phosphorylation following a WNT3A time course. β-catenin protein levels increased in the control cells. CK1γ3 knockdown modestly suppressed WNT3A CM-induced β-catenin stabilization. With simultaneous knockdown of CK1γ1/2/3, stabilization of β-catenin was significantly impaired as compared with control siRNAs (Fig. 5, *C* and *D*). Finally, knockdown of CK1γ3 and CK1γ1/2/3 decreased phosphorylation of LRP6 at T1479 and S1490 compared with the control knockdown (Fig. 5, *C*, *E* and *F*).

### CK1γ3 inhibition impairs activation of WNT signaling

**Figure 6:**
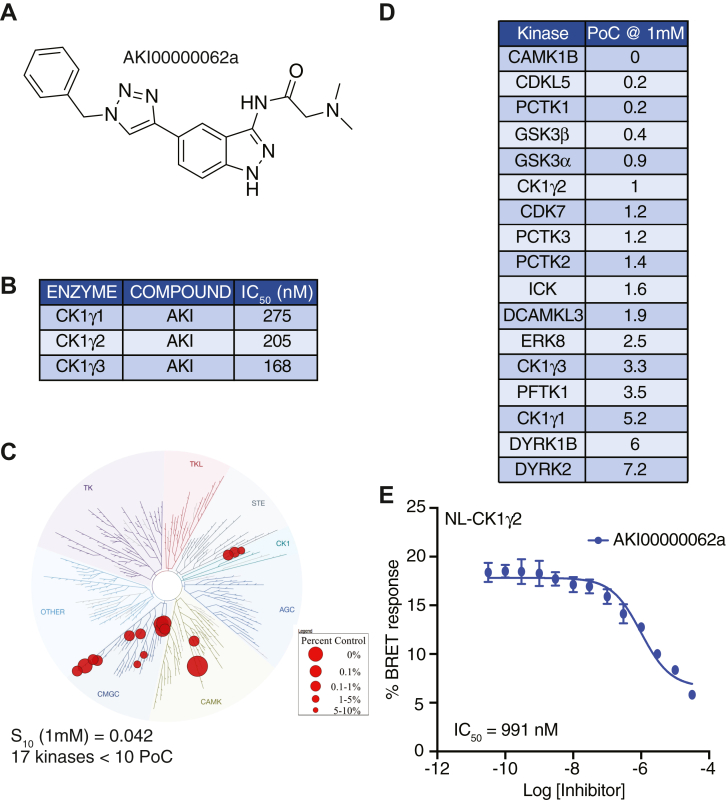
**Summary of data for AKI00000062a.***A*, structure of AKI00000062a. *B*, enzymatic profiling of AKI00000062a *versus* CK1γ family kinases. *C*, kinome tree representation of kinases with PoC <10 when AKI00000062a was profiled at 1 μM *versus* 403 WT kinases. *D*, specific kinases (n = 17) with PoC <10 in kinome-wide profiling. *E*, Nanoluc-CK1γ2 NanoBRET curve, and corresponding IC_50_ value for AKI00000062a. CK1γ, casein kinase 1γ; PoC, Percent of Control.

To complement the genetic study of CK1γ family within the WNT pathway, we sought CK1γ chemical inhibitors. Molecular tool compounds that target the larger CK1 family of kinases have been studied within WNT signaling in the past (49). However, given the multiple positive and negative roles of various CK1 family members in the pathway, and the lack of selectivity for most tool compounds, the results have been inconclusive (50, 51). We leveraged a library of modestly selective kinase inhibitor tool compounds from the University of North Carolina-Structural Genomics Consortium, including a 5-substituted indazole with previously characterized inhibition activity for CK1 (52). To further characterize the lead candidate, we sent AKI00000062a (Fig. 6, *A* and *B*) (referred to as AKI throughout) for kinome-wide profiling against 403 WT kinases at DiscoverX at 1 μM. This compound demonstrated modest selectivity, with a calculated S_10_(1 μM) = 0.042, corresponding to 17 kinases with a Percent of Control (PoC) <10 at 1 μM (Fig. 6*C*). GSK3α and GSK3β were among those kinases potently inhibited as well as all three CK1γ kinases (Fig. 6*D*). To confirm this, we collected the corresponding enzymatic assay activity for all three CK1γ family members and found AKI to inhibit all three enzymes with IC_50_ values ≤275 nM (Fig. 6*B*). Finally, a cellular target engagement assay (NanoBRET) was used to measure the potency of enzymatic inhibition for CK1γ2 in live cells. CK1γ1 and CK1γ3 NanoBRET tools are not yet available. We observed modest suppression of CK1γ2 in the cell-based system: IC_50_ = 991 nM (Fig. 6*E*).

**Figure 7:**
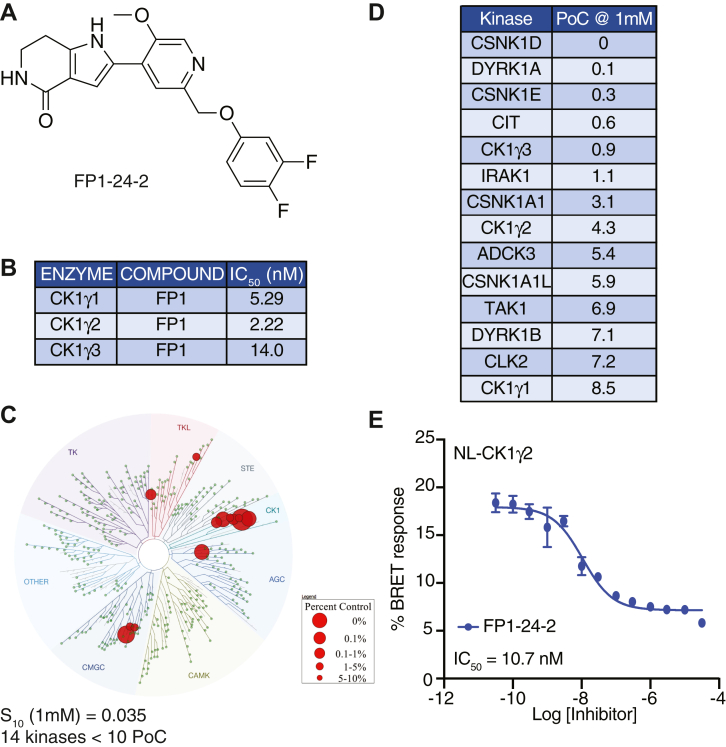
**Summary of data for FP1-24-2.***A*, structure of FP1-24-2. *B*, enzymatic profiling of FP1-24-2 against CK1γ family kinases. *C*, kinome tree representation of kinases with PoC <10 when FP1-24-2 was profiled at 1 μM *versus* 403 WT kinases. *D*, specific kinases (n = 14) with PoC <10 in kinome-wide profiling. *E*, Nanoluc-CK1γ2 NanoBRET curve and corresponding IC_50_ value for FP1-24-2. CK1γ, casein kinase 1γ; PoC, Percent of Control.

Because the AKI compound also targeted GSK3β, we turned to the literature for an alternative chemotype with published potency against the CK1γ kinase subfamily (49). Amgen released a series of pyridyl pyrrolopyridinones as potent and selective CK1γ inhibitors (49). We synthesized one of the published leads (compound 13), furnishing FP1-24-2 with >95% purity (Fig. 7*A*) (referred to as FP1 throughout) (49). We first confirmed the activity of this compound *versus* all three CK1γ enzymes (IC_50_ values <15 nM, Fig. 7*B*). While Ambit KINOMEscan data at 1 μM were included as supporting information in the original publication, kinome-wide selectivity data were lacking (49). We sent FP1 for kinome profiling against 403 WT kinases at DiscoverX at 1 μM. FP1 demonstrated good selectivity with an S_10_(1 μM) = 0.035 (53), corresponding to 14 kinases with a PoC <10 at 1 μM (Fig. 7*C*). Among the potently inhibited kinases are all three CK1γ family members, CK1δ, CK1ε, and CK1α (Fig. 7*D*). NanoBRET was used to test the enzymatic (Fig. 7*B*) and binding (Fig. 7*D*) assay activities for the CK1γ2 kinase in cells (54). Potency was maintained in cells with an IC_50_ of 10.7 nM (Fig. 7*E*).

**Figure 8:**
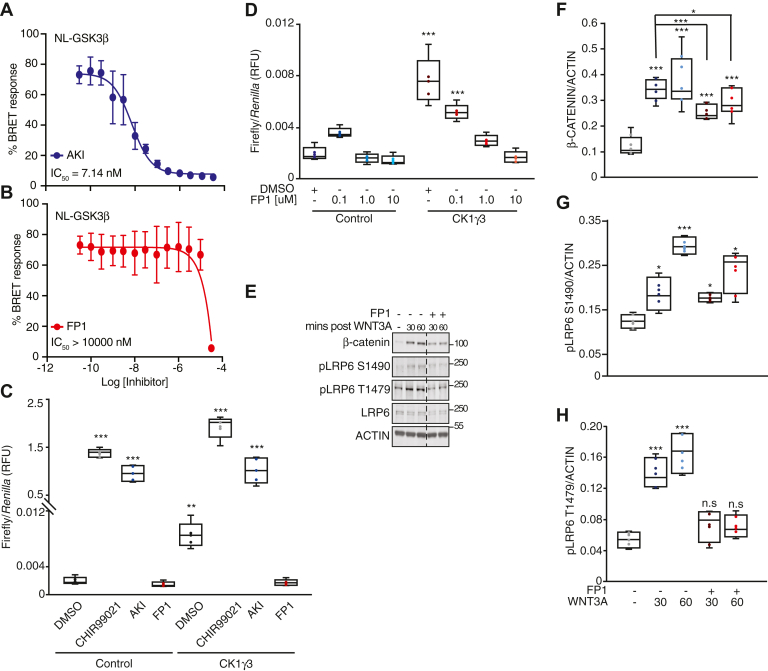
**CK1γ inhibition suppresses β-catenin stabilization and LRP6 phosphorylation.***A* and *B*, Nanoluc-GSK3β NanoBRET curve and corresponding IC_50_ value for AKI and FP1, respectively. *C*, HEK293T B/R cells were transfected with the indicated construct for 14 h and then treated with 10 μM of the indicated compound for 18 h and analyzed by luciferase assay. *D*, HEK293T B/R cells were transfected with the indicated construct for 14 h, treated with DMSO, 0.1, 1.0, or 10 μM of FP1 for 18 h, and then analyzed by luciferase assay. *E*, RKO cells were pretreated with FP1 or DMSO for 1 h, treated with WNT3A ligand for 30 or 60 min, and then analyzed by W. blot. *Dashed line* represents cropped blot for removal of redundant untreated sample. *F*–*H*, quantification of W. blot from *E*, β-catenin (*F*), pLRP6 S1490 (*G*), and pLRP6 T1479 (*H*) across four replicates, normalized to actin. For all panels: ∗∗∗*p* < 0.0005, ∗∗*p* < 0.005, and ∗*p* < 0.05 and are compared to DMSO control unless otherwise indicated. All biological replicates are represented in box-and-whisker plots, with the *boxes* representing the median and the interquartile range. All biological replicates are averaged across three technical replicates. Statistical significance was evaluated by Student’s *t* test. AKI, AKI00000062a; CK1γ, casein kinase 1γ; DMSO, dimethyl sulfoxide; FP1, FP1-24-2; GSK3β, glycogen synthase kinase 3β; HEK293T, human embryonic kidney 293T cell line; W. blot, Western blot.

The kinome-wide profiling of AKI demonstrated potent inhibition of GSK3α and GSK3β; FP1 did not inhibit GSK3α and GSK3β. Using the GSK3β in-cell NanoBRET assay, we found that AKI potently engages GSK3β in cells with an IC_50_ = 7.14 nM (Fig. 8*A*). AKI was significantly more potent on GSK3 kinases than on CK1γ2. In contrast, FP1 was inactive up to 10 μM in the GSK3β NanoBRET assay (Fig. 8*B*). As a control, APY69, validated as active in the GSK3 NanoBRET assay by Promega, was included in both the CK1γ2 and GSK3β NanoBRET assays. APY69 had an IC_50_ = 0.23 nM in the GSK3β NanoBRET assay and was inactive in the CK1γ2 NanoBRET assay. To confirm that AKI inhibits GSK3β, we compared it in a BAR assay with the GSK3β inhibitor, CHIR99021 (Fig. 8*C*) (55). Both CHIR99021 and AKI robustly activated the BAR reporter, as compared with dimethyl sulfoxide and FP1.

Finally, we tested whether FP1 impacted CK1γ3 activation of WNT signaling in a BAR assay. We overexpressed CK1γ3 and treated with increasing doses of FP1 and observed a decrease in BAR activity (Fig. 8*D*). In agreement with this, treatment of cells with FP1 and WNT3A CM resulted in diminished β-catenin stabilization and phosphorylation of LRP6 at site S1490, as compared with the dimethyl sulfoxide control-treated cells (Fig. 8, *E*, *F* and *G*). Importantly, when cells were treated with FP1 and then exposed to WNT3A, LRP6 phosphorylation at T1479 did not increase (Fig. 8, *E* and *H*).

## Discussion

Protein kinases govern information flow though cellular signaling networks and ultimately impact all cell biology. Their exceptional druggability and centrality within human disease networks has supported decades of research and therapeutic development (56). However, attention to kinases has not been evenly spread across the protein class. Recently, the National Institutes of Health Illuminating the Druggable Genome consortium identified 162 of the most understudied “dark” kinases (21). In this study, we characterize three of these “dark” kinases: CK1γ1, CK1γ2, and CK1γ3. Through unbiased protein–protein proximity network derivation and a functional evaluation across a small focused panel of transcriptional reporters, we connect the CK1γ family to the β-catenin-dependent WNT signaling pathway. Unexpectedly we found that the CK1γ3 family member is uniquely active within β-catenin-dependent WNT signaling. All three family members co-complex with both β-catenin-dependent and β-catenin-independent WNT machinery. Furthermore, the CK1γ family activates β-catenin-dependent transcription in the presence of exogenous LRP6 overexpression. CK1γ3 appears particularly potent in its activity as it activates β-catenin-dependent transcription in the absence of exogenous WNT3A ligand or LRP6 overexpression. We show through siRNA-based silencing and small-molecule inhibitors that functional redundancy likely exists within the CK1γ family, as CK1γ3 suppression alone had minimal impacts on WNT signaling. Beyond connections to the WNT pathway, the proximity networks, functional data, and molecular probes offer a deep resource for the kinase community.

Defining proximity networks for this subfamily of understudied kinases provides insight into their cellular functions. Identification of core WNT signaling components, both β-catenin dependent and β-catenin independent, further establishes the CK1γ kinase subfamily as WNT signaling components. Many of the identified proximal proteins localize to the plasma membrane, which is consistent with the reported localization of the CK1γ family, possibly because of palmitoylation of their C terminus (19). One interesting protein identified as abundant and proximal to all three family members is ZDHHC8, a palmitoyltransferase with disease connections to schizophrenia (57). In *Drosophila*, the ZDHHC8 ortholog palmitoylates Scribble, which is a key component of the planar cell polarity pathway and has been shown to negatively regulate WNT/β-catenin signaling in human cell lines (58, 59, 60). It will be important to determine if ZDHHC8 is responsible for palmitoylation and membrane localization of the CK1γ family and reciprocally if CK1γ regulates ZDHHC8 activity or localization. A second interesting and abundant discovery across all three family members is PIK3R3. This regulatory subunit of class 1a PI3K binds phosphorylated tyrosine residues to signal downstream of receptor tyrosine kinases and cytokine receptors (61). PI3K–AKT signal transduction is among the most frequently activated and functionally important pathway in cancer (62). Whether and how CK1γ activity influences PI3K signaling and biology is exciting and warrants testing. Broadly speaking, interrogation of kinase proximity networks like those presented for CK1γ offer a powerful resource for kinase substrate prediction. Notably, however, proximity-based networks have caveats that must be considered, including: (1) the approach is exquisitely sensitive to overexpression artifacts, (2) rigorous probabilistic scoring approaches using appropriate controls are needed to reveal true positives, (3) as with all of proteomics, the absence of identification does not mean absence within the sample but rather below the level of detection; and (4) proximity networks do not identify direct binding relationships. For kinases, motif enrichment queries and integration with phosphoproteomic data can seed experiments for the identification of direct kinase substrates (44).

The observed physical and functional connections to β-catenin-dependent and β-catenin-independent WNT signal transduction are unsurprising. Previous studies demonstrated that CK1γ1 activated β-catenin-dependent WNT signaling with co-overexpression of LRP6 or DVL (19, 20). Importantly, our data replicate these results (Fig. 2, *C* and *D*). Also similar to our results, without co-overexpression, the authors observed minimal activation of WNT signaling with CK1γ1 overexpression (19). Subsequent studies to further articulate the role of the larger CK1γ family in WNT signaling have been lacking until now, including loss-of-function characterizations. One of the surprises of this work is the family unique ability of CK1γ3 to activate β-catenin-dependent transcription in the absence of exogenously added WNT3A ligand or co-overexpression of LRP6. Interestingly however, the C59 inhibitor experiment establishes that CK1γ3 requires an autocrine loop of WNT signaling to impact β-catenin-dependent transcription (Fig. 2*E*). Together, these data build upon prior work to support a role for the CK1γ family and specifically CK1γ3 as important regulators of WNT signaling.

Loss-of-function studies for the CK1γ family within the WNT pathway have not been reported, perhaps because of modest effect sizes. Functional redundancy within the WNT signaling pathway is well acknowledged, and indeed our data support redundancy for the CK1γ family (63, 64, 65). This work and previous studies have shown that CK1δ, CK1ε, and CK1γ can phosphorylate LRP6 (19, 20). In our siRNA-based loss-of-function studies, knock down of all three CK1γ family members was required to observe maximal suppression of the WNT pathway (Fig. 5). Future studies using CRISPR genetic knockouts are needed across each of the CK1 kinase subfamilies. siRNA-based silencing is incomplete, which complicates data analyses, particularly for catalytic proteins like kinases. Coupled with the unique CK1γ3 gain-of-function WNT effects, the functional redundancy evident in the loss-of-function studies raises interesting questions of physiological relevance. It is possible that mRNA overexpression of CK1γ3 in cancer may yield a WNT hyper-responsive cell state, not unlike RNF43 mutations and WNT addiction in pancreatic ductal adenocarcinoma. It is also likely that family unique expression of the CK1γ kinases across tissue and cell types may yield physiologically important differences in WNT sensitivity. Future loss-of-function genetic studies in developmental model systems are needed.

Beyond genetics, we evaluated two tool chemical inhibitors for the CK1 family. The AKI compound also targeted GSK3β, which complicates the study of CK1γ in WNT signaling (Fig. 6). The other compound, FP1, did not affect GSK3β activity but suffered lack of specificity within the CK1 subfamily (Fig. 7). FP1 also targets CK1δ/ε, as well as CK1α, which governs WNT signaling through phosphorylating β-catenin within the destruction complex (Fig. 7). However, FP1 holds value as a research tool when studying proximal events in signalosome formation.

The highly conserved nature of the CK1γ subfamily makes the unique function of CK1γ3 activation of WNT signaling intriguing and raises the question why CK1γ1 and CK1γ2 do not activate WNT signaling with the same potency. Structurally, the CK1γ subfamily is similar with one major exception—CK1γ3 has a 33 amino acid insertion close to the C-terminal tail (Fig. 1*A*). Five β-sheets on the C terminus of the CK1γ subfamily proteins create an open crescent moon shape (66). However, in CK1γ3, the 33 amino acid insertion creates an additional short β-sheet within an intrinsically disordered region (IDR) (66, 67). This β-sheet shifts the C-terminal structure so that the β-sheet lies in an opening of the crescent moon shape (66). In addition, IDRs allow the same peptide sequence to interact differently with different functional outcomes and can have a high number of post-translational modifications (67, 68). The functional implications of the altered structure and the role the IDR plays in CK1γ3 have not been evaluated. Studies that delete the 33 amino acid insertion are necessary to fully understand the functional and mechanistic implications.

Illuminating understudied proteins, particularly kinases, is critical to furthering our understanding of several signaling cascades. This work highlights the need to understand the role these understudied kinases play in signaling pathways and disease states. Full characterization of the understudied kinases presents a previously untapped pool of therapeutic targets to treat a variety of human diseases and improve patient outcome.

## Experimental procedures

### Cell lines and tissue culture

All cells were cultured at 37 °C and 5% CO_2_. The following cells were obtained from American Type Culture Collection (ATCC): HEK293T/17 (human fetus; catalog no.: CRL-11268), RKO (human, gender not available; catalog no.: CRL-2577), Lcells (mouse male; catalog no.: CRL-2648), and WNT3A-expressing Lcells (mouse male; catalog no.: CRL-2648). All cells were grown in Dulbecco's modified Eagle's medium with 10% fetal bovine serum. Each cell line was tested for mycoplasma contamination and passaged no more than 20 passages from the original ATCC stock.

### WNT3A CM

CM was collected as described by ATCC. Briefly, WNT3A and control Lcells were grown to 100% confluency before fresh media were added conditioned for 48 h and then collected.

### Lysis buffers

Radioimmunoprecipitation assay (RIPA) lysis buffer contains 10% glycerol, 50 mM Tris–HCl, pH 7.4, 150 mM sodium chloride (NaCl), 2 mM EDTA, 0.1% SDS, 1% Nonidet P-40, and 0.2% sodium deoxycholate. TAP lysis buffer contains 10% glycerol, 50 mM Hepes (pH 8), 150 mM NaCl, 2 mM EDTA, and 0.1% Nonidet P-40. Triton lysis buffer contains 10% glycerol, 50 mM Tris–HCl (pH 7.4), 150 mM NaCl, 2 mM EDTA, and 1% Triton X-1000.

### Cloning

mT constructs (UBC-mT-V5-CONSTRUCT) were generated by LR multisite recombination (Life Technologies; catalog no.: 11791019; comes with LR Clonase II enzyme mix) utilizing the PEL system (69). CK1γ3 kinase-dead mutants were generated using PCR mutagenesis and cloned into pDONR223 digested with BsrGI using Gibson Assembly Master Mix (New England Biolabs; catalog no.: E2611).

### CK1γ3 K72A (AAG to GCG) primers

CK1γ3 gib A—Forward: 5′-CTT TTT TAT AAT GCC AAC TTT GTA CAA AAA AGT TGG CAT GGA GAA CAA GAA GAA GGA CAA GG

CK1γ3 K72A gib A—Reverse: 5′-GCTCCAGCGCGATGGCCAC

CK1γ3 K72A gib B—Forward: 5′-GTGGCCATCGCGCTGGAGC

CK1γ3 gib B—Reverse: 5′-TTT CTT ATA ATG CCA ACT TTG TAC AAG AAA GTT GGA CTA CTT GTG TCT CTG GAT TGT CTT CC

### Immunoblotting

Standard W. blot techniques were utilized and performed as previously described (15, 43). Briefly, cells were lysed in RIPA lysis buffer, flash frozen on dry ice, and then cleared at high speed for 10 min at maximum speed. Protein concentration was determined by a bicinchoninic acid assay following kit specifications. Samples were balanced, and NuPAGE 4× SDS loading buffer (Thermo Fisher Scientific; catalog no.: NP0007) containing DTT was added to each sample and heated for 10 min at 70 °C. Samples were run on a NuPAGE 4 to 12% Bis–Tris Protein Gel (Thermo Fisher Scientific; catalog no.: NP0321) and then transferred to a nitrocellulose membrane. The membrane was then blocked and incubated overnight with primary antibody at 4 °C with rotation, washed with Tris-buffered saline with Tween-20, incubated with secondary antibody, washed with Tris-buffered saline with Tween-20, and imaged. Images were taken using a LiCOR Odyssey CLx imager and quantified with LiCOR software (LiCOR Biosciences). All antibodies were used at a concentration of 1:1000, with the exception of loading controls, which were used at 1:10,000. All primary antibodies utilized are as follows: β-catenin (BD Biosciences; catalog no.: 163510), LRP6 (Cell Signaling Technology; catalog no.: 3395), phospho LRP6 S1490 (Cell Signaling Technology; catalog no.: 2568), phospho LRP6 T1479 (Life Technologies; catalog no.: PA564736), AXIN1 (Cell Signaling Technology; catalog no.: 3323), GSK3β (BD Biosciences; catalog no.: 610201), CK1γ1 (Abcam; catalog no.: ab190260), CK1γ2 (Abcam; catalog no.: ab64829), CK1γ3 (Abcam; catalog no.: ab116310), DVL3 (Santa Cruz Biotechnology; catalog no.: sc-8027), CELSR2 (Cell Signaling Technology; catalog no.: 47061), V5 (Thermo Fisher Scientific; catalog no.: R960-25), and actin (Santa Cruz Biotechnology; catalog no.: sc-47778). Secondary antibodies were used at a concentration of 1:20,000 and were as follows: IRDye 800CW Goat antimouse IgG (LI-COR Biosciences; catalog no.: 925-32210), IRDye 680LT goat antimouse IgG (LI-COR Biosciences; catalog no.: 925-68020), IRDye800CW goat anti-rabbit IgG (LI-COR Biosciences; catalog no.: 925-32211, IRDye 680LT goat anti-rabbit IgG (LI-COR Biosciences; catalog no.: 925-68021), and IRDye 680LT Streptavidin (LI-COR Biosciences; catalog no.: 926-68031).

### Transcriptional reporter assays

All luciferase reporter assays were performed as previously described (15, 43). Briefly, cell lines stably expressing the BAR-Firefly luciferase reporter and TK-Ren luciferase were used and transfected with either RNAiMAX (Life Technologies; catalog no.: 13778150) for 60 h in loss-of-function experiments and Lipofectamine 2000 (Life Technologies; catalog no.: 11668019) for 36 h in gain-of-function experiments. In both loss-of-function and gain-of-function experiments, treatments (WNT3A CM or drug) were added 18 h before harvesting cells. Conditions were plated in triplicate, and *Renilla* normalized values were averaged across triplicates to yield the data presented and standard error. Each assay was repeated in biological triplicate, unless otherwise stated. Firefly luciferase and the *Renilla* control were detected using the Promega Dual-Luciferase Reporter Assay System per the manufacturer’s protocol (Promega; catalog no.: E1960). Plates were read on the Victor Nivo plate reader (PerkinElmer).

### siRNA knockdown

All siRNAs were obtained from Life Technologies (Thermo Fisher Scientific). Cell lines were used and transfected with RNAiMAX (Life Technologies; catalog no.: 13778150) and for 72 h before W. blot analysis. Sequences are listed in Table S3.

### Real-time quantitative PCR

Total RNA was extracted from RKO and HT1080 cells using Invitrogen PureLink RNA mini kit (Life Technologies; catalog no.: 12183025) following the manufacturer’s manual. The AXIN2 and NKD1 primers are described (43). All other primers were designed using the National Center for Biotechnology Information’s Primer-BLAST platform. Primer sequences are listed in Table S3. Before reverse transcription, RNA was quantified using a Nanodrop One (Thermo Fisher Scientific), and 1 μg of RNA was used to generate complementary DNA with the iSCRIPT Clear kit (Bio-Rad; catalog no.: 170-8891). For the quantitative RT–PCR, PowerUP SYBR Green (Thermo Fisher Scientific; catalog no.: A25741) was used, and data were analyzed on a QuantStudio 5 Real Time PCR machine (Applied Biosystems). ΔCT values were normalized to housekeeping gene RPL13a.

### Affinity pulldowns

Cells were incubated for indicated time with 50 mM biotin, washed 3× in cold PBS, then lysed in RIPA lysis buffer, flash frozen on dry ice, then thawed, and incubated on ice for 30 min. Lysates were then sonicated for 20 s in 5 s pulses. Samples were then high speed cleared, and protein concentration was determined by bicinchoninic acid. About 50 μl of bead slurry per sample was washed 5× in RIPA lysis buffer and then incubated with lysate at 4 °C with rotation for either 1 h for W. blot analysis or overnight for MS analysis. Beads were then washed 5× with RIPA lysis buffer. For W. blot analysis, samples were then eluted in a 1:1:1 mixture of 1 M DTT, 4× SDS loading buffer, and RIPA lysis buffer and heated at 70 °C for 10 min.

### Affinity purification and MS

Following the affinity pulldown (described previously), beads were washed 2× with RIPA, 2× with TAP lysis buffer, and 3× with 50 mM ammonium biocarbonate buffer. About 1 mg of RapiGest SF surfactant (Waters Corporation; catalog no.: 186001861) was resuspended in 200 μl of 50 mM to give a 0.5% RapiGest solution. Streptavidin beads were resuspended in 100 μl of 0.5% RapiGest and vortexed. About 1 M DTT in ammonium biocarbonate was added to the sample for a final concentration of 5 mM, and samples were heated at 60 °C for 30 min. After allowing samples to cool to room temperature, chloroacetamide was added to each sample for a final concentration of 15 mM and incubated in the dark for 20 min at RT. The samples were then centrifuged at 400*g* for 2 min at RT to remove the supernatant containing protein from the streptavidin beads. The supernatant was transferred to a new tube, and 2.5 μg of MS-grade trypsin was added to each sample. The samples were incubated at 37 °C overnight, and then HCl was added to the sample at a final concentration of 250 mM and incubated for 45 min at 37 °C to deactivate the trypsin. Pierce C18 spin columns (Thermo Fisher Scientific; catalog no.: 89870) were placed in a 2 ml centrifuge tube, the column was activated by adding 200 μl of 100% acetonitrile (ACN) and centrifuged at 3000*g* for 2 min. Columns were equilibrated by adding 200 μl of 0.5% TFA and centrifuged at 3000*g* for 2 min. This step was then repeated. The sample was resuspended in 200 μl of 0.5% TFA, then loaded onto the column, and centrifuged for 5 min at 1000*g*. The sample was reloaded onto the column and centrifuged a second time for 5 min at 1000*g*. The column was washed twice with 200 μl of 5% ACN/0.5% TFA and centrifuged for 2 min at 3000*g*. The samples were eluted by adding 50 μl 70% ACN and centrifuged for 5 min at 1000*g*. A second elution step was performed by adding 50 μl 70% ACN and centrifuged for 2 min at 3000*g*. Following a C18 clean up, an ethyl acetate clean up was performed. The sample was resuspended in 100 μl of 0.1% TFA, then 1 ml of water saturated ethyl acetate was added to each sample, vortexed, and centrifuged at maximum speed for 5 min. The upper layer of ethyl acetate was removed. This process was repeated for a total of three times. Samples were then dried down in the speed vac and resuspended in 25 μl of 98/2 buffer A (water + 0.1% formic acid [FA])/buffer B (ACN + 0.1% FA).

### MS data acquisition

Trypsinized peptides from 30 min biotin-treated cells were separated *via* reverse-phase nano-HPLC using an RSLCnano Ultimate 3000 (Thermo Fisher Scientific). The mobile phase consisted of water + 0.1% FA as buffer A and ACN + 0.1% FA as buffer B. Peptides were loaded onto a μPAC Trapping column (PharmaFluidics) and separated on a 200 cm μPAC column (PharmaFluidics) operated at 30 °C using a 100 min gradient from 2% to 25% buffer B, followed by a 20 min gradient from 25% to 35% buffer B, flowing at 300 nl/min. MS analysis was performed on an Orbitrap Eclipse (Thermo Fisher Scientific) operated in data-dependent acquisition mode. MS1 scans were acquired in the Orbitrap at 120 k resolution, with a 250% normalized automated gain control target, auto maximum injection time, and an *m/z* scan range of 375 to 1500. Both the linear ion trap and the Orbitrap were used for MS2 scans. MS2 targets with a charge of +2 or +3 and >90% precursor fit at either an *m/z* 0.8 or *m/z* 0.4 -wide isolation window were fragmented with by collision-induced dissociation at 35% collision energy and scanned in the linear ion trap at the widest window width that passed the thresholds. All remaining MS2 targets with charge from +2 to +6, >5e4 intensity, and >10% precursor fit at isolation widths of *m/z* 1.6 , *m/z* 0.8 , or *m/z* 0.4 wide were fragmented with higher-energy collision dissociation at 30% collision energy and scanned in the orbitrap at 15 k resolution at the widest window width that passed the thresholds. MS2 automated gain control targets and maximum injection times were set to standard and auto for their respective analyzers. Dynamic exclusion was set to 60 s. Acquisition was performed with a 2.7 s cycle time. For 120 min biotin-treated samples, the same method was used except the following: peptides were separated on a 50 cm μPAC column using a 115 min gradient from 2% to 25% B followed by a 5 min gradient from 25 to 30% B, MS1 scans were acquired at 240 k resolution, and an *m/z* 375 to 2000 scan range. All MS2 scans were acquired in the linear ion trap using an *m/z* 0.7-wide isolation window and fragmented with higher-energy collision dissociation at 35% collision energy. Acquisition was performed with a 1.0 s cycle time.

### MS data processing

Raw MS data files were processed by MaxQuant (version 1.6.17.0) (70) using the UniProtKB/SwissProt human canonical sequence database (20,428 entries; downloaded on August 2019) (71). To facilitate comparison to another 88 bait experiments performed within our laboratory, the files were processed simultaneously with these raw files. The following parameters were used: specific tryptic digestion with up to two missed cleavages, fixed carbamidomethyl modification, variable modifications for protein N-terminal acetylation, methionine oxidation, match between runs, label-free quantification, 4.5 ppm precursor mass tolerance in the main search, 20 ppm fragment mass tolerance for Orbitrap MS2 scans, and 0.5 Da fragment mass tolerance for ion trap MS2 scans. The protein accepted false discovery rate (FDR) was set to 1% and determined using the target-decoy strategy implemented by MaxQuant. Only unique peptides were used for protein quantification because of the high tryptic peptide overlap between CK1γ1, CK1γ2, and CK1γ3. Baits were assigned fraction numbers >1 away from each other to enable match-between-runs within replicates but not across different baits. Prey proteins were filtered for high-confidence physical interactions and proximal proteins using SAINTexpress (version 3.6.3) (36, 37) with the following thresholds: ≥2 unique peptides, AvgP ≥0.7, and Bayes FDR ≤0.05. SAINT scoring was performed separately for 30 min and 120 min biotin-treated experiments using their respective control runs. About 10% of interactions were scored by the CompPASS WD score (https://github.com/dnusinow/cRomppass), validated WNT-related proteins from the literature, and hits from published siRNA and CRISPR screens for WNT pathway regulation (38, 39, 40, 41, 42). The criteria for screen hits were as follows (also described in Table S1): ≤0.01 FDR for Lebensohn *et al.*, twofold change and greater for Biechele *et al.*, greater than or less than twofold change in one cell line with two single siRNAs for Madan *et al.*, and twofold change and greater for at least two siRNAs for Major *et al.* (39, 40, 41, 42). Gene Ontology annotations were extracted with the org.Hs.eg.db R package, and overrepresentation analysis was performed with ClusterProfiler using default parameters https://bioconductor.org/packages/release/data/annotation/html/org.Hs.eg.db.html. Accessed Nov 2021 (72).

### Source of compounds

FP1-24-2 was prepared as previously described (49). Details are provided in the next section. AKI00000062a was donated to North Carolina-Structural Genomics Consortium by AbbVie.

### Synthesis of 2-(2-((3,4-difluorophenoxy)methyl)-5-methoxypyridin-4-yl)-1,5,6,7-tetrahydro-4*H*-pyrrolo[3,2-*c*]pyridin-4-one (FP1-24-2)

To a flask was added 2-bromo-1-(2-((3,4-difluorophenoxy)methyl)-5-methoxypyridin-4-yl)ethan-1-one (26.3 mg, 1.0 equivalent, 70.7 μmol), piperidine-2,4-dione (9.59 mg, 1.2 equivalent, 84.8 μmol), and ammonium acetate (21.8 mg, 4.0 equivalent, 283 μmol) in ethanol (283 μl, 0.25 M), and the reaction was stirred at room temperature for 16 h. The reaction was concentrated *in vacuo* and purified by preparative HPLC (10–100% MeOH in H_2_O (+0.05% TFA) to yield the desired product 2-(2-((3,4-difluorophenoxy)methyl)-5-methoxypyridin-4-yl)-1,5,6,7-tetrahydro-4*H*-pyrrolo[3,2-*c*]pyridin-4-one as an off-white solid (1.9 mg, 7%).

^1^H NMR (400 MHz, MeOD-*d*_4_): δ 8.30 (s, 1H), 7.77 (s, 1H), 7.22 to 7.13 (m, 2H), 6.99 (ddd, *J* = 3.0, 6.7, 12.4 Hz, 1H), 6.85 to 6.80 (m, 1H), 5.08 (s, 2H), 4.09 (s, 3H), 3.57 (t, *J* = 7.0 Hz, 2H), 2.97 (t, *J* = 7.0 Hz, 2H). ^13^C NMR (101 MHz, MeOD-*d*_4_): δ 169.0, 152.1, 150.1, 140.8, 134.4, 130.3, 127.7, 119.3, 118.48, 118.46, 118.29, 118.28, 115.5, 111.74, 111.70, 111.68, 111.64, 110.5, 105.7, 105.5, 72.0, 57.0, 41.7, 23.0. ^19^F NMR (376 MHz, MeOD-*d*_4_): δ −138.23 – −138.37 (m), −150.69 (dddd, *J* = 3.3, 6.6, 10.2, 20.8 Hz). LC–MS: expected mass for [M + H]^+^ (C_20_H_18_F_2_N_3_O_3_), *m/z* 386.12; found, *m/z* 386. LC–MS and ^1^H NMR: >95%.

### CK1γ family enzymatic assays

Eurofins' kinase enzymatic radiometric assays were carried out at the *K*_*m*_ = ATP in dose–response (9-pt curve in duplicate) for each CK1γ kinase listed in Figures 6*B* and 7*B*. Details related to the substrate used, protein constructs, controls, and assay protocol for each kinase assay are included on the Eurofins website: https://www.eurofinsdiscoveryservices.com/.

### Kinome-wide profiling

The *scan*MAX assay platform was used to assess the selectivity of FP1-24-2 and AKI00000062a at 1 μM at Eurofins DiscoverX Corporation. As described previously, this commercial assay platform screens against 403 WT human kinases in binding assays and provides PoC values (73). All WT kinases that demonstrated PoC values <10 are plotted on the respective kinome trees in Figures 6*C* and 7*C*. These same WT kinases with PoC value <10 are tabulated in Figures 6*D* and 7*D*.

### NanoBRET assays

HEK293T cells (hypotriploid, female, fetal) were purchased from ATCC. These cells were grown in Dulbecco’s modified Eagle’s medium (Gibco) supplemented with 10% (v/v) fetal bovine serum (Corning). They were incubated in 5% CO_2_ at 37 °C and passaged every 72 h with trypsin and not allowed to reach confluency. Constructs for NanoBRET measurements of CK1γ2 (NLuc-CK1γ2) and GSK3β (NLuc-GSK3β) were kindly provided by Promega. NanoBRET assays were carried out as described previously (74). Preferred NLuc orientations, both N-terminal in this case, are indicated in parentheses after each construct. Assays were carried out in dose–response as described by the manufacturer using 0.5 μM of tracer K-10 for CK1γ2 and 0.063 μM of tracer K-8 for GSK3β. Two biological replicates were executed with two technical replicates to produce the graphs with error bars shown in Figures 6*E*, 7*E*, 8, *A* and *B*. Tracer titration curves for these kinases that motivated our tracer selection and working concentration can be found on the Promega website.

## Data availability

The MS proteomics data have been deposited to the ProteomeXchange Consortium *via* the PRIDE partner repository with the dataset identifier PXD032138 (75).

## Supporting information

This article contains supporting information.

## Conflict of interest

The authors declare that they have no conflicts of interest with the contents of this article.

## Supporting information

Table S1 Protein-protein interaction networks

Table S2 Identifying overlap in published screens

Table S3 siRNA sequences and Primers
